# Assessment of the Risk of Orthorexia Nervosa and Attitudes Towards Their Own Bodies among Amateur and Professional Soccer Players

**DOI:** 10.5114/jhk/201971

**Published:** 2025-05-26

**Authors:** Wiktoria Staśkiewicz-Bartecka, Grzegorz Zydek, Małgorzata Magdalena Michalczyk, Wojciech Mroszczyk, Marek Kardas

**Affiliations:** 1 Department of Food Technology and Quality Evaluation, Medical University of Silesia in Katowice, Katowice, Poland.; 2 Institute of Sport Sciences, Jerzy Kukuczka Academy of Physical Education in Katowice, Katowice, Poland.; 3 Department of Sport Nutrition, Jerzy Kukuczka Academy of Physical Education in Katowice, Katowice, Poland.; 4 Bruk-Bet Termalica Nieciecza, Soccer Club, Żabno, Poland.

**Keywords:** body image, eating disorders, team sports, athletes

## Abstract

Orthorexia Nervosa, although not recognized as an official mental disorder, is a behavior characterized by a pathological focus on healthy eating. The purpose of the study was to assess the risk of Orthorexia Nervosa and attitudes toward one's own body among amateur and professional soccer players. The survey was administered among 137 male soccer players, both amateur and professional, from different Polish leagues. A mixed method survey (direct and electronic) was applied, using the Düsseldorfer Orthorexia Scale and the Body Esteem Scale to assess Orthorexia Nervosa risk and attitudes towards one's own body. The survey was administered between February and April 2024. The results showed that professional soccer players had higher satisfaction with their bodies compared to amateurs. Approximately 27% of the participants were at risk of Orthorexia Nervosa, regardless of their sports level. The analysis showed a weak but statistically significant correlation between the risk of Orthorexia Nervosa and scores on the upper body strength subscale. The results suggest that professional athletes may have a better relationship with their bodies, which may result from a more systematic and conscious approach to training and diet. At the same time, it is confirmed that Orthorexia Nervosa is present among soccer players. The study underscores the need for awareness of the risk of Orthorexia Nervosa among soccer players and points to the importance of support at both the amateur and professional levels.

## Introduction

Professional athletes are more susceptible to developing eating disorders (EDs) compared to the general population (Neglia, 2021). Restrictive diets frequently cause prolonged food limitation, which can lead to EDs such as bulimia nervosa (BN), anorexia nervosa (AN), binge eating disorder (BED), and other specified eating disorders (OSFED) (Mancine et al., 2020). Statistics on the prevalence of these disorders among athletes are alarming. For instance, a survey conducted by Flatt et al. (2021) involving 3509 professional athletes revealed that 74% of them experienced overeating, 26% admitted to vomiting, and 50% reported engaging in self-starvation. EDs are dangerous and can negatively affect athletic success and the physical and mental health of athletes (Mancine et al., 2020).

### Orthorexia Nervosa

People with orthorexia nervosa (ON) focus more on the quality of food than the quantity, leading to highly restrictive diet that may cause nutritional deficiencies, malnutrition, health issues, and a lower quality of life (Koven and Abry, 2015). Although there have been efforts to define diagnostic criteria for orthorexia, none have been officially recognized. Consequently, orthorexia is not currently classified as a formal mental disorder and is not listed as an eating disorder in the two main classification systems for mental illnesses. European countries utilize the International Statistical Classification of Diseases and Related Health Problems (ICD-10), while the United States follows the Diagnostic and Statistical Manual of Mental Disorders (DSM-5). Both classification systems offer detailed descriptions of various EDs, including AN, BN, and other related conditions, aiding professionals in accurate diagnosis and treatment planning for those affected. However, neither system currently includes specific criteria for ON (APA, 2013; WHO, 2019). In practice, however, ON is often considered as OSFED or seen as a new cultural manifestation of AN. This means that while ON does not have its formal diagnostic category, it is frequently classified under OSFED, which includes various atypical EDs that do not meet the criteria for more specific diagnoses like AN or BN (Jenkins et al., 2021).

### EDs vs. Type of Physical Activity

While soccer is the primary focus of this study, it is important to recognize that EDs are prevalent across a variety of sports, not just team-based ball sports like soccer. Different sports pose different risks for EDs, often depending on the specific physical demands and cultural expectations associated with the sport. For instance, sports that emphasize aesthetic appearance, such as gymnastics, figure skating, and ballet, tend to have higher incidences of EDs due to pressures to maintain a lean physique for performance and judgment purposes (Godoy-Izquierdo et al., 2023; Torstveit et al., 2008). Similarly, weight-class sports like wrestling, boxing, and martial arts place athletes under significant pressure to meet strict weight categories, which can encourage unhealthy weight control practices (Barnes et al., 2016; Mancine et al., 2020). Even endurance sports such as long-distance running and cycling have been linked to a higher risk of EDs due to the emphasis on leanness for improved performance (Ibáñez-Caparrós et al., 2023; Sundgot-Borgen and Torstveit, 2010). By considering these various sports contexts, we can see that the risk of EDs is influenced by both the type of physical activity and the specific expectations placed on athletes in those sports. Consequently, understanding the broader landscape of EDs across different sports not only enhances the relevance of this research, but also situates the findings within a wider context, demonstrating that the risks posed by EDs are not limited to soccer or ball sports, but are a significant concern throughout the sporting world (Texidor-Batlle et al., 2021).

Although soccer is not considered high-risk, there is increasing evidence that altered eating behaviors and EDs symptoms are common, affecting about 1/4 to 2/3 of soccer players (Gouttebarge et al., 2015). Uriegas et al. (2021), in their study with college athletes, found that an average of 17.7% of athletes were at risk of ON, with a higher prevalence among women. A similar trend was seen in ball sport athletes, with approximately 24% of women and 14% of men at risk of ON (Urigeas et al., 2021).

### EDs vs. Sports Level

The level of competition is another risk factor for EDs, with athletes at higher competitive levels displaying more signs of body dissatisfaction and EDs compared to those at lower levels or non-athletes. Athletes competing at higher levels face more rigorous training schedules, greater pressure to achieve an ideal physique or specific weight, and are more likely to have perfectionist tendencies (Picard, 1999). Athletes not only endure very high training loads because of the unique demands of their sport, but many also dedicate their entire lives to elite sports. This often results in an excessive commitment to the norms and values of the elite sports culture, with little influence from outside the sports environment (Cash and Deagle, 1997; Hughes and Coakley, 2016).

Fochesato et al. (2021) discovered that the prevalence of EDs was linked to the level of competition, with elite athletes being at the greatest risk. Similarly, Werner et al. (2013) found that athletes competing at higher levels showed symptoms of EDs more often than those at lower performance levels or recreational athletes. In the available scientific literature, research has focused on the general concept of EDs, and there is a lack of studies assessing the prevalence of orthorexic behavior in athletes considering their various sports levels.

### EDs vs. Gender

While EDs were traditionally viewed as a problem primarily affecting female adolescents, recent studies indicate that men may account for up to 25% of ED cases (Ahlich et al., 2018). EDs are not exclusive to any gender, and over time, diagnostic criteria have been revised to include men. Among college-aged men, common behaviors include binge eating (7.9%), excessive exercise (4.4%), fasting (4%), self-induced vomiting (2.7%), and abuse of laxatives or diuretics (1.6%) (Dakanalis et al., 2016). Various factors increase the risk of EDs in men, such as psychological influences, societal expectations related to gender roles, media depictions of dieting and fitness, the association of muscularity with masculinity, social media messages about body image, and significant fluctuations in body mass (Griffiths et al., 2015).

In the context of gender and sports identity, ED behaviors may go unnoticed as problematic or even be encouraged. Seeking help may be hindered by feelings of shame, the stigma around mental health treatment in athletes, internal conflicts between masculine norms and athlete identity, or the stereotype that eating disorders are primarily a female issue (Eichstadt et al., 2020).

Men practicing team sports are usually excluded from the high-risk population for EDs development. This exclusion can be questioned because current information about EDs in sports is insufficient, particularly for male team sports players. Some studies warn about the potential underdiagnosis of EDs in men and it is not yet known whether being part of a team acts as a protective factor or as a risk factor for EDs development (Baldó Vela and Bonfanti, 2019).

ON, although affecting both men and women, may develop differently depending on gender, especially in male-dominated sports such as soccer. Research suggests that men may be more susceptible to developing ON due to social pressures associated with achieving an idealized, muscular physique, which is often seen as a marker of masculinity (Brodock et al., 2024). This phenomenon can be further exacerbated by the culture of sport, where physical strength and fitness are essential for performance, and appearance norms may lead to an excessive focus on healthy eating. In soccer , where players face significant pressure to maintain peak physical condition and body weight for optimal performance, ON may emerge as a response to these demands. Although ON is more commonly observed in women, gender differences in soccer highlight the need for further research into how men may be exposed to ON in different ways than women and how these differences could influence the risk of eating disorders among soccer players (While et al., 2020).

### EDs vs. Body Image

Body image is often described as a complex psychological concept concerning perceptions and experiences of one's own body, including its physical appearance along with attitudes and perceptions (Cash, 2004). The attitudinal component of body image refers to subjective appraisal, such as satisfaction or dissatisfaction with one's body appearance and related beliefs and attitudes, which can influence behavior, emotions, and thinking. The perceptual component, on the other hand, includes the mental representation of one's own body or parts of it (Pauzé et al., 2021). Body image disorders can involve both attitudinal and perceptual components, and their symptoms can vary across these dimensions. For example, negative attitudes can manifest as dissatisfaction with one's appearance, feelings of anxiety or shame related to one's body, distorted beliefs about one's appearance, an excessive focus on one's appearance, or body avoidance. Concerning satisfaction with appearance, conflicting data suggest that ON may be associated with both lower and higher satisfaction with appearance (Pauzé et al., 2021; White et al., 2020). Nevertheless, the fact that many other attitudes related to body image are also correlated with ON suggests that there is some relationship between ON and certain attitudes towards body image (Plichta et al., 2019). However, the methodological and psychometric limitations of these studies, as well as the paucity of research in this area, underscore the need for further research.

Soccer, one of the world’s most popular sports, predominantly appeals to a male audience. Soccer players often experience body dissatisfaction due to social and media pressures, expectations of athletic performance, and comparisons with other athletes (Baldó Vela et al., 2022). Social media’s promotion of idealized male beauty and physique standards contributes to this dissatisfaction, leading to stress, lowered self- esteem, and lifestyle modifications, including dietary changes. The ongoing pressure to achieve ideal physical standards may increase the risk of EDs among soccer players (Godoy-Izquierdo et al., 2019; Staśkiewicz-Bartecka and Kardas, 2024). Although current literature reviews indicate that there are few studies assessing the risk of EDs and attitudes towards body image in soccer players, there are no studies specifically examining the risk of ON in this group of athletes.

Therefore, the present study aimed to evaluate the risk of ON and attitudes towards body image among amateur and professional male soccer players. It sought to identify possible correlations between body perception, the risk of ON, and the level of athletic competition. It was hypothesized that 1) orthorexic behavior would be more common among professional soccer players compared to amateurs; 2) professional soccer players would be characterized by higher satisfaction with their bodies; and 3) there would be a relationship between attitudes towards body image and the risk of ON in soccer players, with those exhibiting negative attitudes about their bodies expected to have a higher risk of ON.

## Methods

### Study Design and Procedures

The survey was administered using a mixed survey method and questionnaire technique from February to April, 2024. A total of 68 male soccer players participated in the direct survey; they were players from clubs competing at the level of the 1^st^ division (one club) and the 2^nd^ division (two clubs). Athletes completed the questionnaire at the club after training, in addition, instruction was provided to the participants before completing the questionnaires to ensure proper understanding and interpretation of the questions and to minimize completion errors. The response rate was 90.67%. The survey was also administered among players from the 5^th^ division (three clubs) electronically, as an indirect survey (computer assisted web interview, CAWI). Due to the lack of infrastructure at sports clubs to conduct the survey, players were given a QR code that redirected them to the survey questionnaire, which is an acceptable method in psychological research. The researchers provided instructions to participants before handing over the QR code to ensure proper understanding and interpretation of the questions and to minimize completion errors. Soccer players completed the survey on the day they received the code. There were 69 male players in this group. The response rate for this group was 89.61%.

The study used purposive sampling, where the sample was chosen to reflect specific characteristics and experiences relevant to the research topic. Key features such as gender, the sport practiced, and the performance level were carefully defined to meet the study's objectives.

Participants in the study were briefed on the study's purpose and assured of its anonymity. They were also asked to agree to the terms of data sharing. Information about informed and voluntary participation was provided at the beginning of the questionnaire. The World Medical Association's Declaration of Helsinki guided the conduct of this study. The survey was approved by the Bioethics Committee of the Silesian Medical University in Katowice (approval code: BNW/NWN/0043-3/641/35/23, date of approval: 22/09/2023) in light of the Law of December 5, 1996 on the Profession of Physician and Dentist (Journal of Laws 2016, item 727).

### Participants

The survey included 137 male players aged 18–36, who were members of clubs competing at various levels of men's soccer in Poland. Players in the 5^th^ division clubs were categorized as amateur athletes (AAs), while those in the 1^st^ and 2^nd^ division clubs were grouped as professional athletes (PAs). The survey was conducted during the first half of the spring round of the 2023/2024 league season, with all players tested within the same timeframe (between the 6^th^ and 8^th^ rounds) to ensure consistent and comparable results.

The inclusion criteria for the study group were as follows: (1) consent from the clubs to conduct the study, (2) voluntary participation and completion of the questionnaire, (3) age 18 or older, (4) male, (5) active status as a club player during the study, (6) no injuries that had prevented training for at least 7 days in the two months prior to the study, (7) proficiency in Polish or English, and (8) no history or current diagnosis of EDs. The exclusion criterion was an incorrectly or incompletely completed questionnaire.

### Research Tools

The study was conducted using a survey questionnaire, which included a metrics section (collecting data on respondents' age, height, body mass, field position, education, chronic illnesses and medications, sources of nutritional information, dietary exclusions, and physical activity outside club training) as well as the Düsseldorf Orthorexia Scale (DOS, PL-DOS) and Body Esteem Scale (BES) questionnaires.

### BMI

The body mass index (BMI) was calculated by dividing the body mass (kg) by the square of the body height (m). These results were then used to compare height-to-weight ratios for the European population against WHO recommendations and guidelines (World Health Organization, 2010).

### E-DOS, PL-DOS

The Düsseldorf Orthorexia Scale (DOS) is a screening tool that assesses orthorexic eating behavior. The DOS shows good internal consistency, good construct validity, and good test-retest reliability (Meule et al., 2020). The ten-item Düsseldorf Orthorexia Scale is a subscale of the Orthorexic Eating Behavior subscale of the longer 21-item DOS, which has a total of three subscales, the other two being the Additive Avoidance subscale and the Mineral Supply subscale. The ten-item DOS is used as a one-dimensional measure to assess and screen ON (Barthels et al., 2015). The ten items are answered on a four-point Likert scale ranging from "definitely not applicable to me" to "definitely applicable to me". There are no reverse items. The maximum number of points to be obtained in this test is 40. According to the interpretation, a total score greater than 30 points indicates the presence of ON, a score between 25 and 29 points indicates the risk of ON, while a total score of less than 25 points indicates the absence of ON (Barthels et al., 2015).

Due to the presence of people of different nationalities in the study group, the current study used a ten-item English-language questionnaire (E-DOS) and a Polish version of the ten-item DOS (PL-DOS). The study showed that the PL-DOS had good reliability to the E-DOS (α = 0.840, ω = 0.840) (Barthels et al., 2015; Brytek-Matera, 2021). For the Düsseldorf Orthorexia Scale, the McDonald's ω coefficients were 0.781 for the English version (E-DOS) and 0.867 for the Polish version (PL-DOS), indicating good internal consistency for both versions of the scale used in our study.

### BES

A survey of respondents' body image was conducted using the Body Esteem Scale (Franzoi and Shields, 1984). The Polish version of the questionnaire was developed by Lipowska and Lipowski (2013). This scale allows assessing the respondents' attitudes towards their own bodies. Due to the presence of people of different nationalities in the study group, the original version of the questionnaire and the Polish-language version were used. The Polish-language version was shown to have good reliability to the English-language version, for the whole tool, with α = 0.93, while for the male group, with α = 0.94 (Franzoi and Shields, 1984; Lipowska and Lipowski, 2013). For the BES, the McDonald's ω coefficients were 0.912 for the English version and 0.978 for the Polish version, indicating excellent internal consistency for both versions of the scale.

The scale is composed of 35 questions that are divided into three subscales which differ based on the respondent's gender. For men, the subscales are physical attractiveness (PA), upper body strength (UBS), and physical fitness (PF). Responses are given on a five-point Likert scale, where "1" represents strong negative feelings, "5" represents strong positive feelings, and "3" is neutral.

The physical attractiveness subscale for men focuses on assessing features that are key to considering a man handsome. It covers a variety of items, including facial features and various parts of the body, such as the hips or feet. While the evaluation of sexual organs has some relevance to this scale, it does not take into account their function or sexual activity. The body strength subscale is based on the assessment of various body parts (such as the arms or chest), as well as their functionality and efficiency, which are relevant to strength and physical activity. Physical fitness, on the other hand, focuses on assessing the endurance and agility of the body (Lipowska and Lipowski, 2013).

To interpret the results, a normative table that includes age categories and stens, specifically designed for evaluating men's scores, was used. This table is divided into six age groups and 10 stens. When the score fell between 1 and 3 on the sten scale, it was considered a low body score; scores ranging from 4 to 7 were considered medium, and scores between 8 and 10 were classified as high (Franzoi and Shields, 1984; Lipowska and Lipowski, 2013).

### Statistical Analysis

Statistical analyses were conducted using Statistica v.13.3 (Stat Soft Poland) and the R package v. 4.0.0 (2020) under the GNU GPL (The R Foundation for Statistical Computing). For quantitative data, mean values and standard deviations (X ± SD) were calculated, while qualitative data were presented using percentage notation. The Shapiro-Wilk test was used to assess normal distribution compliance. Differences between amateur and professional soccer players were evaluated using the Student's *t*-test for two parametric groups, analysis of variance (ANOVA) for three or more parametric groups, the Mann-Whitney U test for two non-parametric groups, and the Kruskal-Wallis test for three or more non-parametric groups.

Pairwise comparisons of body weight measurements were made using the Durbin Conover test.

To explore the relationship between DOS and BES scores, the Spearman's rho coefficient was applied. This coefficient allows for assessing the strength and direction of the association between two ordinal variables, specifically the risk score for developing ON and the BES score.

A value of *p* < 0.05 was used as a criterion for statistical significance.

## Results

### Sample Characteristics

A total of 137 soccer players participated in the study, following the application of the inclusion and exclusion criteria. The players were divided into two groups based on their level of competition: the first group (n = 69) comprised amateur athletes (AA), while the second group (n = 68) consisted of professional athletes (PA). The players were of various nationalities: 156 Poles, 3 Ukrainian, 2 Slovak, 2 Spanish, 1 English, 1 Macedonian, 1 Georgian, and1 Japanese. Five soccer players suffered from chronic diseases (2: hypothyroidism, 1: Hashimoto's, 1: hypertension, 1: asthma); four athletes were taking constant medication (Euthyrox for hypothyroidism and Hashimoto's, Berodual for asthma).

**Figure 1:**
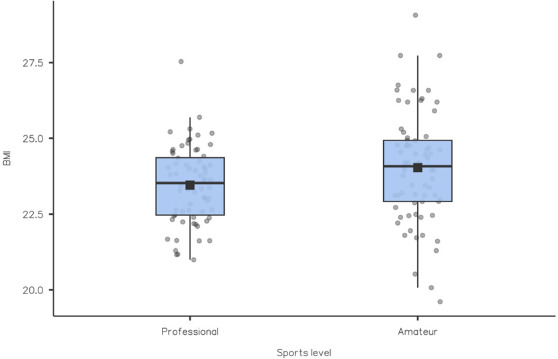
Distribution of BMI values among amateur athletes (n = 69) and professional athletes (n = 68).

Athletes were also asked about their lowest and highest body mass in adulthood, as well as what they thought was their ideal body mass. There was a statistically significant difference between amateur and professional soccer players in their response to the question about the highest body mass (*p* = 0.025). In addition, a statistically significant difference was shown between the highest (*p* < 0.001) and the lowest (*p* < 0.001) body mass and current body mass in the entire study group. No such significant difference was shown between the current body mass and what athletes considered to be the ideal body mass (*p* = 0.691) (Table 1). Details of the respondents' BMI with regard to their sports level are shown in Figure 1.

**Table 1:** Characteristics of the study group, including the current, lowest, highest, and considered ideal body mass of soccer players (n = 137).

	Age [years] (X ± SD)	Height [cm] (X ± SD)	Body mass [kg] (X ± SD)	BMI [kg/m^2^] (X ± SD)
Total (n = 137)	26.7 ± 5.18	180.2 ± 6.39	77.2 ± 1.60	23.7 ± 1.60
AA (n = 69)	27.4 ± 5.27	179.3.1 ± 5.76	77.4 ± 8.11	24.0 ± 1.83
PA (n = 68)	26.1 ± 5.05	181. 1 ± 6.88	77.1 ± 7.90	23.5 ± 1.27
*p*-value	0.154	0.088	0.514	0.041*
	Body mass CURRENT (X ± SD)	Body mass LOWEST (X ± SD)	Body mass HIGHEST (X ± SD)	Body mass IDEAL (X ± SD)
Total (n = 137)	77.2 ± 1.60	71.7 ± 9.53	80.2 ± 11.60	76.3 ± 9.66
AA (n = 69)	77.4 ± 8.11	71.9.1 ± 6.97	82.1 ± 10.41	76.8 ± 7.32
PA (n = 68)	77.1 ± 7.90	71.9 ± 11.61	78.2 ± 12.50	75.9 ± 11.59
*p*-value	0.514	0.504	0.025*	0.813
	CURRENT – HIGHEST *p* < 0.001*; CURRENT – LOWEST *p* < 0.001*; CURRENT – IDEAL *p* = 0.691			

X – Average; SD - Standard deviation; AA- amateur athletes; PP- professional athletes, * p < 0.05

The main sources of nutritional knowledge for PAs were a dietician (n = 48; 70.59%) and the Internet (n = 45; 66.18%), followed by coaches (n = 16; 23.53%), scientific literature (n = 14; 20.59%), and other athletes (n = 18; 26.48%). In the AA group, athletes most often indicated the Internet as the source of nutritional knowledge (n = 46; 66.67%), followed by a dietician (n = 14; 20.29%), the coach (n = 17; 24.64%), and other athletes (n = 16; 23.18%). There was a correlation between the sports level and the source of nutritional knowledge, with the PA group more likely to identify a nutritionist (*p* < 0.001) and scientific literature (*p* = 0.010).

### Risk of ON

According to the interpretation of the DOS questionnaire results, it was shown that 27% (n = 37) of athletes had the risk of ON, while 6.6% (n = 9) suffered from ON. There was no significant relationship between the risk or presence of ON and the performance level (*p* = 0.572) (Table 2).

**Table 2:** Summary of ON risk estimation (DOS) (n = 137).

DOS	AA (n = 69) n (%)	PA (n = 68) n (%)	Total (n = 137) n (%)	*p*-value
No risk < 25	47 (68.12)	44 (64.72)	91 (66.40)	0.572
Risk 25–29	19 (27.54)	18 (26.48)	37 (27.00)	
Presence > 30	3 (4.34)	6 (8.70)	9 (6.60)	
	AA (n = 69) (X ± SD)	PA (n = 68) (X ± SD)	Total (n = 137) (X ± SD)	*p*-value
Points	20.6 ± 6.73	23.0 ± 4.59	21.8 ± 5.87	0.572

X – Average; SD - Standard deviation; AA- amateur athletes; PP- professional athletes, * p < 0.05

**Figure 2:**
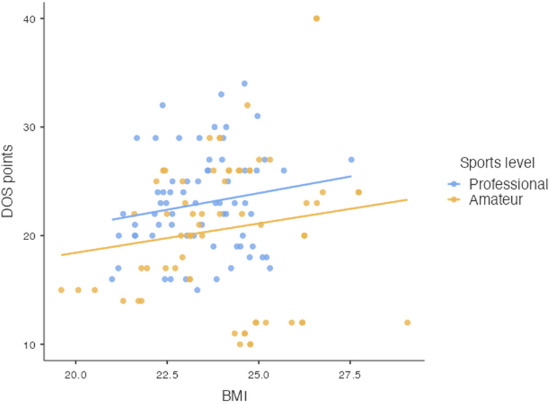
Scatter plots showing the sum of scores obtained in the DOS with the BMI, by the sports level of players (n = 137).

However, a significant relationship was found between the performance level and the average score obtained on the DOS scale (*p* = 0.027). Nevertheless, no significant relationship was observed between the performance level and the BMI (*p* = 0.110) (Figure 2).

### Attitude towards One’s Own Body

The analysis of the BES questionnaire results showed that soccer players had moderate body ratings in the categories of PA, UBS, and PA. Additionally, significant differences were found between amateur and professional athletes. When analyzing the mean values according to the sten scale, professional athletes had higher average sten scores across all subscales (*p* = 0.002; *p* = 0.008; *p* < 0.001). Likewise, professional soccer players scored higher in all subscales as interpreted by the BES (*p* = 0.014; *p* < 0.001; *p* < 0.001). Detailed results are presented in Table 3.

**Table 3:** Assessment of the athletes' body attractiveness according to the number of sten and BES interpretation (n = 137).

	Total (n = 37)			AA (n = 69)				PA (n = 68)				*p*-value	
PA [sten] X ± SD	6.13 ± 2.39			5.52 ± 2.36				6.75 ± 2.28				0.002*	
UBS [sten] X ± SD	6.00 ± 2.07			5.38 ± 2.25				6.63 ± 1.65				0.008*	
PC [sten] X ± SD	5.41 ± 1.94			4.86 ± 2.12				6.52 ± 2.16				<0.001*	
Assessment of the attractiveness subscale:	Low	Medium	High		Low	Medium	High		Low	Medium	High		*p*-value
PA n (%)	25 (18.2)	72 (52.6)	40 (29.2)		19 (27.5)	34 (49.3)	16 (23.2)		6 (8.8)	38 (55.9)	24 (35.3)		0.014*
UBS n (%)	21 (15.3)	82 (59.9)	34 (24.8)		19 (27.5)	37 (53.6)	13 (18.8)		2 (2.9)	45 (66.2)	21 (30.9)		<0.001*
PC n (%)	23 (16.8)	90 (65.7)	24 (17.5)		22 (31.9)	39 (56.5)	8 (11.6)		1 (1.5)	51 (75.0)	16 (23.5)		<0.001*

PA: physical attractiveness; UBS: upper body strength; PF: physical fitness; X: average; SD: standard deviation; AA: amateur athletes; PP: professional athletes, * p < 0.05

**Figure 3:**
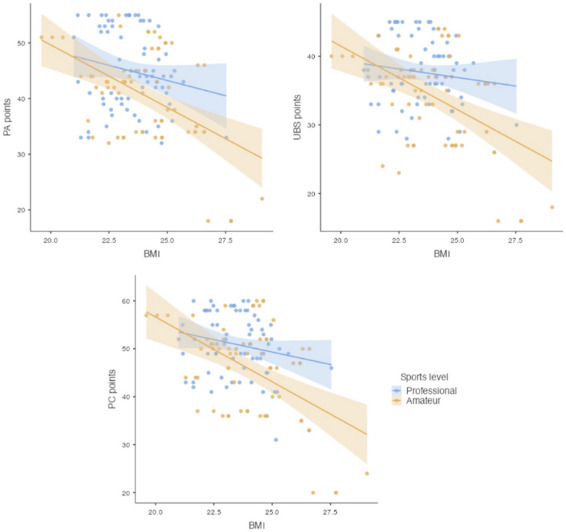
Scatter plots depicting the aggregate scores from BES across the subscales: Physical Attractiveness (PA), Upper Body Strength (UBS), and Physical Fitness (PF), to BMI values, considering the sports level of participants.

The differences between groups of athletes were also analyzed based on the BMI interpretation and self-assessment of body attractiveness. A correlation was found between BMI values and self-assessment in all subscales: PA (*p* = 0.032), UBS (*p* = 0.002), and PF (*p* < 0.001). The total BES scores across all three subscales in relation to BMI values are illustrated in Figure 3.

Professional athletes, on average, gave the highest ratings to the "Physical Fitness" (4.49 points) and "Health" (4.5 points) aspects, while the lowest ratings were given to "Hips" (3.84 points), "Feet" (3.78 points) and "Body Hair" (3.76). Amateurs, on the other hand, on average rated the "Reflex" (4.14 pts.) and "Body Structure" (4.16 pts.) aspects highest, and "Body Smell" (3.38 pts.), "Nose" (3.32 pts.) and "Feet" (3.41 pts.) lowest.

We also examined the relationship between interpretations of DOS scores and BES scores on all three subscales, and found a significant, albeit weak, correlation between the risk of developing ON and UBS subscale scores in soccer players (*p* = 0.049). However, there were no significant correlations between the risk of developing ON and scores on the PA subscale (*p* = 0.344) or the PC subscale (*p* = 0.713). These results are detailed in Table 4.

**Table 4:** Relationship between the DOS score and BES.

DOS		BES			Spearman's ρ
	Low n (%)	Medium n (%)	High n (%)	*p*-value	
	PA				
No risk	19 (20.9)	47 (51.6)	25 (27.5)	0.344	0.081
Risk	4 (10.8)	21 (56.8)	12 (32.4)		
Presence	2 (22.2)	4 (44.4)	3 (33.3)		
	UBS				
No risk	17 (18.7)	56 (61.5)	18 (19.8)	0.049*	0.204
Risk	1 (2.7)	23 (62.2)	13 (35.1)		
Presence	3 (33.3)	3 (33.3)	3 (33.3)		
	PC				
No risk	14 (15.4)	64 (70.3)	13 (14.3)	0.713	0.032*
Risk	6 (16.2)	22 (59.5)	9 (24.3)		
Presence	3 (33.3)	4 (44.4)	2 (22.2)		

PA: physical attractiveness; UBS: upper body strength; PF: physical fitness; * p < 0.05

## Discussion

ON defined as the obsessive desire to eat only healthy foods, is becoming an increasingly recognized problem among athletes who are under constant pressure to maintain optimal physical form. Such behavior can lead to a range of negative health effects, including nutritional deficiencies, psychological disorders, and impaired athletic performance.

Soccer players may be particularly vulnerable to ON due to the strong emphasis on physical fitness and body aesthetics, which can lead to an unhealthy attitude towards food and an obsessive focus on their image. In addition, physical ideals are often promoted in society and the media, which can add to the pressure. Our study focused on assessing this risk and analyzing how soccer players viewed their bodies, which could provide valuable clues to understanding how this group could be better supported in maintaining both physical and mental health.

In light of the study's objectives and hypotheses, our analysis focused on understanding how ON risk and attitudes towards one's body affected amateur and professional soccer players. We expected that orthorexic behavior would be more prevalent among professional soccer players, who were also characterized by higher satisfaction with their bodies. In turn, a negative attitude towards one's self-image could increase the risk of ON, suggesting a significant relationship between self-perceived attractiveness and the risk of developing ON.

The results of the study partially support the hypotheses. The results indicate that professionals were more satisfied with their bodies, which might be due to a more systematic and conscious approach to training and diet, which are integral to their professional activities. However, surprisingly, the results did not conclusively confirm that ON was more prevalent among professionals, suggesting that other factors or the discipline itself may be a predisposing factor in the development of ON. The study found that 27% of subjects had a risk of developing ON, while 6.6% already had this disorder.

Orthorexic behaviors have been associated with a need for perfect self-representation and an addiction to exercise (Surała et al., 2020). Furthermore, such behaviors have been observed in individuals highly focused on maintaining a healthy lifestyle, including yoga enthusiasts, fitness devotees, and frequent social media users (Myrissa et al., 2023). However, within the sporting community, findings regarding the prevalence of ON are inconsistent (de Oliveira et al., 2017).

In a study by Surała et al. (2020) analyzing ON among athletes, the results indicated that the incidence of ON was independent of gender, age, and the type of athletic activity, although was associated with body mass, the BMI, bone mineral content, lean soft tissue, and visceral fat in men (Surała et al., 2020). In another study conducted among college athletes, the risk of ON and EDs was assessed, examining gender differences and dependence on the type of sport disciplines practiced. The results showed a 17.7% incidence of ON. Approximately 20.9% of participants showed a risk of EDs, with clear gender differences and dependence on the type of sport practiced (Pratt et al., 2022). Our results do not confirm the prevalence of ON at this level. The differences may be explained by a different survey instrument used.

In a study of Staśkiewicz-Bartecka et al. (2025), elite soccer players showed high prevalence of ED risk, with more than half of the players showing potential symptoms and one-third at risk of ON. Higher body fat content was associated with increased ED risk and lower self-esteem, while muscle mass showed no significant correlation with ED attitudes. In contrast, no significant association was found between ON and body composition (Staśkiewicz-Bartecka et al., 2025). These findings were confirmed in another study involving youth soccer players (Zydek et al., 2025).

The study of Myrissa et al. (2023) highlights significant insights into ON among athletes, addressing the psychometric efficacy of instruments like the Teruel Orthorexia Scale (TOS) and the Eating Habits Questionnaire (EHQ). It illustrates the prevalence and variance of ON behaviors between elite and recreational athletes, revealing that elite athletes tend to have higher scores in healthy orthorexia and are more knowledgeable about healthy eating, likely due to their structured training and dietary regimes (Myrissa et al., 2023). A study by Karaağaç et al. (2022) on exercise dependence and EDs among college athletes and students (non-athletes) showed that college athletes were at higher risk of exercise dependence and showed stronger orthorexic tendencies compared to non-athletes. These results suggest that sports experience is related to both exercise dependence and orthorexic tendencies (Karaağaç et al., 2022). The results of our study are opposite to those described above and indicate that there is no relationship between the performance level and ON risk.

The results of the study by Staśkiewicz and Kardas (2024) regarding attitudes towards one's own body and the risk of EDs among amateur and professional soccer players indicate the complexity of the problem at both the amateur and professional levels. Although the risk of EDs was comparable in both groups, BES interpretations revealed that professional athletes rated their bodies higher than amateurs. This may suggest that professionals have a better relationship with their own bodies, which is supported by our results (Staśkiwicz-Bartecka and Kardas, 2024). However, for that study, soccer players from the 4^th^ division were classified as professionals, while those from the 5^th^ division were classified as amateurs, thus the difference in their sports levels was not as significant as in our study.

The study by Oliveira et al. (2018), including individuals practicing different sport disciplines, showed that dissatisfaction with body image was observed in all groups, but its prevalence varied by sport, with higher values for athletes participating in sports that promoted slimness or a certain body mass, such as synchronized swimming, rowing, gymnastics and all combat sports with weight categories (Oliveira et al., 2018). This finding was confirmed in another analysis of athletes' body weight perceptions, as even when athletes fell within the ideal standard of relative body fat for athletes in these respective sports, they showed dissatisfaction with their body mass, presenting a need to reduce it (Domingues and Carmo, 2021). In our study, there were no differences between the current body mass of soccer players and what they considered to be the ideal body mass, indicating that athletes participating in the study were satisfied with their body mass.

In a study by Kong and Harris (2015), the body dissatisfaction and symptoms of EDs among athletes at both elite and amateur levels were compared. The findings revealed that athletes, irrespective of their competition level, who engaged in sports that valued leanness, reported higher levels of body dissatisfaction and were more prone to exhibit symptoms of EDs. Those competing in higher leagues experienced more body dissatisfaction and a greater prevalence of EDs symptoms, regardless of their sport type, a contrast not seen in amateur or non-athletic participants. Additionally, it was noted that over 60% of elite athletes faced pressure from their coaches about their body image, across both types of sports (Kong and Harris, 2015). Our results showed an inverse relationship between the issue of self-perception and the sports level.

Although ON and other EDs, such as AN and BN, share certain characteristics, such as excessive control over food intake and an obsessive focus on body weight, there are important differences between them, particularly in the context of sports (Mancine et al., 2020). ON is primarily focused on the quality of food rather than the quantity, distinguishing it from AN, where the goal is calorie restriction, or bulimia, which combines episodes of overeating with compensatory behaviors like self-induced vomiting (Koven and Abry, 2015). In sports, ON can be more difficult to identify, as behaviors such as restrictive diets and meticulous food control are often viewed as healthy habits and even encouraged by coaches and peers. In contrast, symptoms of AN and BN, such as drastic weight loss or extreme purging behaviors, are more noticeable and may raise greater concern. Additionally, in ON, the key issue is an obsession with the "purity" of food, which can lead to psychological and physical consequences related to nutrient deficiencies, as well as exercise addiction. This combination of strict diets and excessive exercise can significantly impact athletic performance. Understanding these subtle differences is crucial for recognizing and effectively intervening with athletes who are at risk of developing various types of EDs (Brodock et al., 2024).

Our survey of amateur and professional soccer players had several strengths. First, the use of standardized survey instruments, such as the Düsseldorf Orthorexia Scale and the Body Esteem Scale, increased the reliability of the results. In addition, the two language versions of the questionnaires allowed for a more complete understanding of the cultural and linguistic influences on participants' responses. In addition, the study included athletes from different performance levels, allowing for a broader comparison and understanding of the problem in different sports contexts. In addition, the very high response rate of soccer players (90.67% for professionals and 89.61% for amateurs) demonstrated the commitment of participants, which increased the representativeness of the results and reduced the risk of oversampling errors.

However, our study has also some limitations. The survey was based on self-report questionnaires, which could lead to errors related to participants' subjective perceptions of their behavior and attitudes. Clear and understandable instructions to participants before the survey increased the likelihood that responses would be well thought out and accurate, which is crucial to the reliability of self-reported data. The survey was cross-sectional, making it impossible to observe changes over time in participants' attitudes and behaviors. In addition, limiting the study to male soccer players only does not allow for an understanding of the problems associated with ON nervosa and body dissatisfaction among female athletes.

## Conclusions

Based on the results of the study, several important conclusions can be drawn about ON risk and attitudes toward one's own body among amateur and professional soccer players.

Professional soccer players are more likely than amateurs to derive knowledge about nutrition from dieticians and the scientific literature, which may suggest a more conscious approach to diet and a healthy lifestyle. Similar levels of ON risk were reported in both groups. About 27% of amateurs and professionals were found to have a risk of developing ON, while 6.6% already suffered from ON. This suggests that other factors, such as the sport practiced, an individual's approach to health, access to balanced sources of nutrition information or personal beliefs about healthy eating, may be more important in shaping the risk of ON than mere involvement in sports at the professional level. The results showed that professional athletes generally evaluated their bodies more positively than amateurs, as manifested by higher average scores on the BES scale in all subscales analyzed. This may be related to the greater attention professionals pay to their physical condition and physical appearance as part of their professional activities. The analysis showed a weak but statistically significant correlation between ON risk and scores on the UBS subscale. This suggests that negative self-perceptions of physical strength may be a risk factor for ON. In contrast, no significant associations were found between ON risk and scores on the PA and PF subscales. The significant differences among the highest, the lowest and current body mass across the study group, and the lack of significant differences between current body mass and the body mass considered ideal, may indicate stability and satisfaction with body mass among the soccer players studied. However, it is worth noting the potential for interventions to support healthy body perceptions, especially in the context of avoiding extreme body mass changes that can lead to health problems.

In conclusion, the results of the study indicate the significance of the risk of developing ON in soccer players. The findings may also be useful for coaches, dietitians, and sports psychologists in developing educational and support programs for athletes at different performance levels.
